# Case-controlled structure validation

**DOI:** 10.1107/S0907444908041085

**Published:** 2009-01-20

**Authors:** Randy J. Read, Gerard J. Kleywegt

**Affiliations:** a Department of Haematology, University of Cambridge, Cambridge Institute for Medical Research, Wellcome Trust/MRC Building, Hills Road, Cambridge CB2 0XY, England; b Department of Cell and Molecular Biology, Uppsala University, Biomedical Centre, Box 596, SE-751 24 Uppsala, Sweden

**Keywords:** protein structures, validation, case-control method, resolution, structural genomics

## Abstract

Although many factors influence the quality of a macromolecular crystal structure, validation criteria are usually only calibrated using one of these factors, the resolution. For many purposes this is sufficient, but there are times when one wishes to compare one set of structures with another and the comparison may be invalidated by systematic differences between the sets in factors other than resolution. This problem can be circumvented by borrowing from medicine the idea of the case-matched control: each structure of interest is matched with a control structure that has similar values for all relevant factors considered in this study. In addition to resolution, these include the size of the structure (as measured by the volume of the asymmetric unit) and the year of deposition. This approach has been applied to address two questions: whether structures from structural genomics efforts reach the same level of quality as structures from traditional sources and whether the impact factor of the journal in which a structure is published correlates with structure quality. In both cases, once factors influencing quality have been controlled in the comparison, there is little evidence for a systematic difference in quality.

## Abstract 2

A case-matched control protocol provides a useful method to compare sets of macromolecular structures.

## 1. Introduction

Structure validation is a blanket term that covers a variety of methods to determine the local and global quality of a macromolecular crystal structure (Kleywegt, 2000 ▶, 2009 ▶). How validation should be carried out depends on how the analysis will be used. For instance, a biologist extracting structural information from the PDB is interested in the absolute quality of a particular structure; if there are several entries to choose from, he or she will not care whether a low-resolution model was determined exceptionally carefully if there is an indifferently determined but higher resolution model of higher absolute quality available.

On the other hand, a structural biologist depositing a structure can only hope to do the best that is possible with the amount of information available for that structure. He or she will probably be happy (as will the database curator evaluating the deposition) if the structure is at least as good as other structures determined using similar amounts of information.

Our analysis concerns this form of relative validation, in which the quality of a structure is evaluated against the quality of comparable structures. This is not only of value for structure deposition, but also for comparing sets of structures determined by different methodologies or in different settings. One particular application is to compare the quality of structures determined as part of structural genomics initiatives with those determined in conventional research settings.

Ideally, the gold standard for structure validation should be the best that could possibly be achieved with the amount of information available or at least the best that can be achieved by the most talented crystallographers using current methods. Setting the baseline for such comparisons would require a tremendous amount of work that is far beyond the scope of this study. Nonetheless, a medium-scale experiment showed that using the right tools it is possible to make substantial improvements in the quality of typical structures (Arendall *et al.*, 2005 ▶) and even an automated protocol can make im­provements in the average structure (Joosten *et al.*, 2009 ▶). Practising crystallographers should thus aim to do better than the average seen so far in comparable structures in the PDB.

### 1.1. Global factors influencing structure quality

There are many possible factors that could plausibly influence structure quality, many of which will be highly correlated with each other. Since the present work was a pilot study, we only attempted to control for a small number of the factors that we believed would be most important.

The quality that can be achieved in a structure determination will be limited by the amount of information that is available. Since the new experimental information comes from the diffraction pattern, the most obvious criterion is the resolution to which the diffraction data have been collected. However, even this criterion is subject to confounding effects, with investigators differing in their choices of the resolution limit to which data should be collected from a particular crystal. In this work, we use the resolution limit reported by the author.

Information comes from other sources as well. For instance, if there is noncrystallographic symmetry with a substantial rotational component (Kleywegt & Read, 1997 ▶) or even just a high solvent content then the effective number of observations per parameter will increase. However, it would be necessary to develop new tools to extract this information from the data available in the PDB. Similarly, if there are multiple crystal forms then the model can be improved by using multi-crystal averaging to clarify the electron density or by transferring structural information from refinement against a high-resolution data set into the model for a lower-resolution data set. Unfortunately, this factor is even more difficult to quantify. Finally, structures determined by molecular replacement using models from higher resolution structures will inherit at least some of their higher quality, depending on the level of sequence identity and thus the amount of rebuilding required. Not all PDB entries record information about the models used for molecular replacement, so it is difficult to rigorously account for this effect. As a surrogate, Brown & Ramaswamy (2007 ▶) defined the ‘similarity index’ of a structure as the number of earlier depositions that belong to the same cluster of proteins sharing at least 50% sequence identity.

The overall quality of a structure is presumably also influenced by other factors. For example, we might expect the average quality of protein structures to change over time (Kleywegt & Jones, 2002 ▶). On one hand, structure-determination methods are steadily improving; on the other, more and more of the structures are being determined by scientists who are primarily biochemists or molecular biolo­gists but not highly trained crystallographers, and larger and often more poorly diffracting systems are tackled. To evaluate the influence of time, we tracked the year of deposition. One might also expect that larger structures will on average be of poorer quality than smaller structures, partly because of data-collection issues (intrinsically weaker diffraction, increased overlap, increased radiation damage) and partly because of the increased burden of manual rebuilding. To evaluate this effect, we tracked the asymmetric unit volume. Technical difficulties caused by effects such as twinning and anisotropy could also limit the quality of the resulting structures, but we did not attempt to investigate these effects. Finally, it is conceivable that the space group or Laue group could influence the structure quality (for example, through differences in packing restrictions or increased uncertainties in unit-cell parameters for lower symmetry space groups), so we looked for any trends influenced by symmetry.

### 1.2. Accounting for the influence of global trends

If we wish to evaluate the quality of a structure against the quality of comparable structures, then we have to be very careful in how we define ‘comparable’. This is a particularly tricky issue when we attempt to compare sets of structures that differ in one factor (such as average size) which may in turn be correlated with other important factors (such as the resolution limit of the diffraction pattern). For instance, we may wish to compare the quality of structures determined recently with those determined 20 y ago. However, over that time period the average size of structures has probably increased and the average resolution might well have changed, so the comparison has to take account of any influence of size and resolution. Similarly, if we wish to compare the quality of structures published in the highest impact journals with those published elsewhere, we have to account for systematic differences in size and resolution. In comparing the quality of structures determined in structural genomics initiatives with conventional structures, we have to consider the size of the structures (probably smaller on average for structural genomics), resolution (structural genomics efforts may apply more stringent criteria to continue a project) and possibly the method of structure solution (structural genomics structures will more frequently be determined by experimental phasing methods).

In developing *PROCHECK*, Laskowski *et al.* (1993 ▶) assumed that resolution was the most important criterion, so that all statistics were reported relative to those for structures at a similar resolution. Recently, Brown & Ramaswamy (2007 ▶) carried out a multivariate statistical analysis to model the impact of a number of factors determining quality on a number of validation measures. However, their analysis assumed that the dependence of the quality metrics on the factors influencing quality was linear. As discussed below, this assumption does not appear to be valid, casting doubt on at least some of the conclusions that they reached.

We wished to avoid making unnecessary assumptions about which factors would turn out to be significant in influencing the expected structure quality or about the functional dependence of average quality on these factors. The method we chose was case-controlled validation, in which every structure being evaluated (‘case’) was matched with one or more examples of control structures that share similar values of the factors that might influence quality. In the first phase, we looked at sets in which some of the factors that might influence quality were varied one at a time, matching controls for all other factors. The second phase built on the understanding of significant factors to find matching controls in looking at two possible correlations with structure quality: the impact factor of the journal in which the structure was published and whether or not the structure was determined within a structural genomics initiative.

### 1.3. Validation criteria

We have explored a number of validation criteria. Firstly, to verify that the matching of cases with controls has succeeded, we looked at the average values of the matching criteria. The conventional *R* and *R*_free_ reported by the authors have been extracted from the PDB-file headers. Other criteria (scores based on the Ramachandran plot, rotamer scores, packing quality *etc*.) were obtained from the Uppsala Electron Density Server (EDS; Kleywegt *et al.*, 2004 ▶), *MolProbity* (Davis *et al.*, 2007 ▶), *PROCHECK* (Laskowski *et al.*, 1993 ▶) and *WhatCheck* (Hooft *et al.*, 1996 ▶). For structures for which experimental data have been deposited, additional results have been extracted from EDS, including the *R* factor from *REFMAC* and scores evaluating the real-space fit of the model to the electron density.

Even the validation criteria can be subject to confounding effects from systematic differences among groups of structures. For example, if different investigators apply different criteria in deciding which poorly or­dered residues to include or omit from the deposited model, there will be a systematic effect on criteria such as the fit to electron density or the quality of the Ramachandran plot that vary locally with the degree of order.

## 2. Matching cases with controls

A relatively simple procedure was used to match cases with controls. For each of the matching criteria, a target was set for differences that would be considered small. In this pilot study, we matched structures by resolution (target difference of 0.1 Å), year of deposition (target difference of 2 y) and asymmetric unit volume (target difference of 10%). Rounds of matching were then carried out. In the first round, each possible control structure was compared with case structures and if the differences in matching criteria were small relative to the target (with a threshold initially set at one fifth of the target difference) the structures were flagged as matched. In subsequent rounds, the target thresholds were increased linearly and an attempt was made to match structures that had not been matched in earlier rounds. Any structures that were not matched by the time that the threshold had increased to five times the target were left out of subsequent comparisons.

As an example, when structural genomics structures were matched with structures determined outside of structural genomics initiatives, 99.5% of the structures were matched by cycle 5, when the threshold was equal to the target difference, and all the structures were matched by cycle 18, when the threshold was 3.6 times the target difference.

## 3. Identifying independent factors that influence structure quality

This study was carried out with protein X-ray crystal structures available in the PDB in December 2007. Elimination of entries with no protein component or with an asymmetric unit volume less than 5000 Å^3^ (probably small peptides) left 38 860 entries. Of these, 23 352 had statistics available in EDS, indicating that structure factors had been deposited and that they were consistent with the coordinates.

### 3.1. Effect of symmetry

For most of the factors we are considering, it is possible to define matching criteria that allow a certain amount of variation. On the other hand, it is difficult to define a numerical measure for similarity between different space groups; for example, if a match cannot be found for a structure in space group *P*312, is *P*622 more similar than *P*2, *P*3, *P*321 or *P*3_1_12? For this reason, we first looked for systematic effects of symmetry on the quality that can be attained in a structure determination to determine whether the space group was an important independent factor.

Table 1 ▶ shows that there are significant differences between space groups in the average resolution, *R* factors and size of the asymmetric unit, although these differences are not large on an absolute scale. However, when structures in one space group are matched with structures in other space groups that diffract to similar resolution, were determined at a similar time and have a similar asymmetric unit volume, then the differences in validation criteria (illustrated by the *R* factors in Table 1 ▶) are negligible.

It would be interesting to establish why the space group can have a systematic effect on factors such as the resolution to which the crystal diffracts. Fortunately, for our case-control study it was not necessary to worry about matching the symmetry if we matched other factors such as resolution. In fact, to avoid the tendency of the matching algorithm to choose isomorphous pairs of structures, in the work discussed below we only matched structures if the space groups were different.

### 3.2. Variation over time

In addition to factors such as the gradual increase in size of structures determined, comparison of structure quality over time has the additional complication that the rate of structure determination is rising exponentially. As a result, for the earlier time points it is not possible to select a significant number of structures over a narrow range of dates.

To cope with this, we took as our basis set the 210 structures that were deposited in the PDB between 1972 and 1987 and that satisfied our inclusion criteria. We then matched these to the structures deposited in subsequent periods, choosing the length of the periods so that substantially more than 210 structures had been deposited and were available for matching. We thus took samples from 1988–1992, 1993–1994 and then annually from 1995. For each time period, we matched the 1972–1987 (case) structures with the later (control) time period and thereby obtained statistics for sets of structures that were comparable in all important factors other than time of deposition.

**Figure 1:**
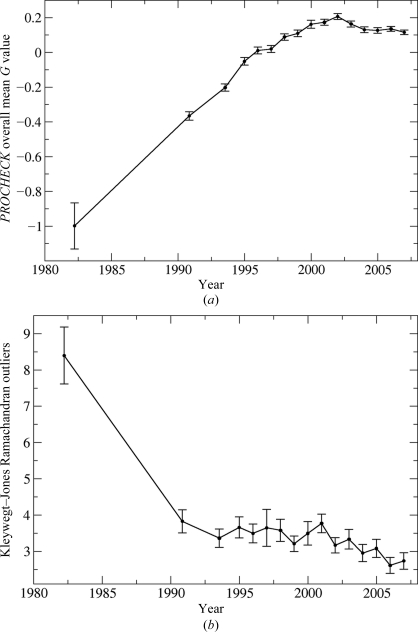
Variation in mean validation criteria over time, comparing the structures deposited from 1972 to 1985 with matched structures deposited over subsequent periods. Error bars in this and subsequent figures indicate the estimated standard uncertainty of the mean, computed as the sample standard deviation divided by the square root of the number of observations. (*a*) Overall average *G* value computed by *PROCHECK* (Laskowski *et al.*, 1993 ▶). (*b*) Percentage Ramachandran outliers determined using the criteria established by Kleywegt & Jones (1996 ▶).

Only a subset of quality measures could be tracked over time, as only a small minority of the earlier structures have associated structure-factor depositions and some of the earlier entries even lack information on the *R* factor. For all the measures of structure quality that could be compared, there was a large improvement between the first two data points (*i.e.* the periods covering 1972–1987 and 1988–1992), at least for the type of structures that were determined in the early years of protein crystallography. Since 1992, there has been a steady but slower improvement in most measures of quality. Fig. 1 ▶(*a*) shows one exception: quality as measured by the overall average *G* value determined by *PROCHECK* (Laskowski *et al.*, 1993 ▶) improved until about 2000, but has levelled off since then. This is probably because the standards established in 1993 are too forgiving. In contrast, Fig. 1 ▶(*b*) shows that the more stringent measure of Ramachandran outliers, as defined by Kleywegt & Jones (1996 ▶), continues to show a gradual improvement in recent years.

### 3.3. Effect of resolution

**Figure 2:**
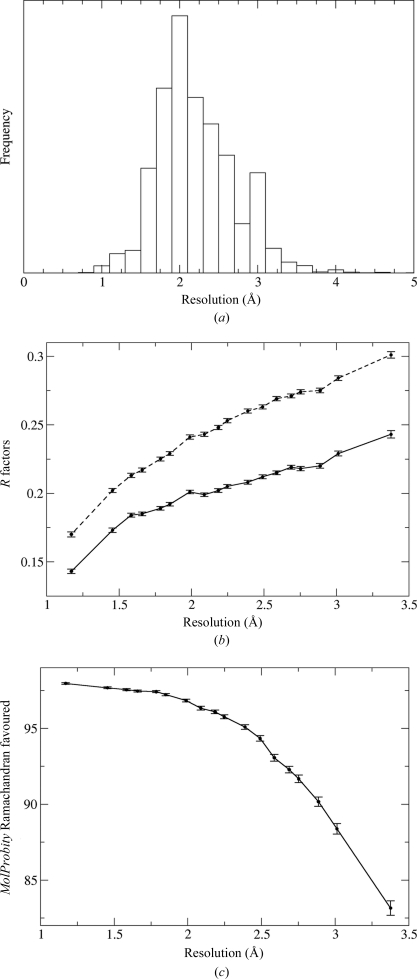
Variation in validation criteria with resolution, comparing structures with a resolution limit from 2.05 to 2.06 Å with matched structures in other resolution ranges. (*a*) Histogram of reported resolution limits over the set of structures considered in this study. There are 33 structures with a resolution lower than 5 Å, but these would not appear on the scale of this histogram. (*b*) *R*_work_ (solid line) and *R*_free_ (dashed line). (*c*) Percentage of residues in favoured regions of the Ramachandran plot, as assessed by *MolProbity* (Davis *et al.*, 2007 ▶).

Comparison of structures with different resolution limits is complicated, like the comparison with time, by the fact that the distribution of resolution limits is far from uniform (Fig. 2 ▶ *a*). 80% of structures in the set we studied diffracted to reported resolutions between 1.6 and 2.8 Å, even though the range extends from 0.54 to 22 Å.

The basis set we chose for comparison was a set of structures from a narrow window at the peak of the distribution, *i.e.* 408 structures determined to a resolution of between 2.05 and 2.06 Å. These were treated as ‘case’ structures and matched with controls from resolution windows chosen to contain significantly more structures. The structures were divided into resolution shells extending over 0.1 Å, except at the low- and high-resolution ends of the range, where shells from 0.54 to 1.3, 1.3 to 1.5, 2.9 to 3.1 and 3.1 to 10 Å were used.

It is not a surprise to see that Figs. 2 ▶(*b*) and 2 ▶(*c*) show a strong dependence of validation criteria on reported resolution. One feature to emphasize is that for many of the criteria the dependence on resolution is strikingly nonlinear. Fig. 2 ▶(*c*) shows one such example: the percentage of residues that fall into the favoured regions of the Ramachandran plot as defined in *MolProbity* (Davis *et al.*, 2007 ▶). If a linear fit were carried out, it would be dominated by the data near 2 Å resolution and predictions from the linear fit at the resolution extremes would be inaccurate.

We suspect that such nonlinearities are responsible for the differences between the conclusions we reach and those reached by Brown & Ramaswamy (2007 ▶). In their work, they carried out a multivariate fit between various validation criteria and factors influencing quality, but the fit was according to a linear model. Therefore, if the multivariate fit is then used to assess the quality of structures that fall outside of the range of the bulk of the data used to define the fit, the prediction of the quality that should be achieved will be inaccurate.

### 3.4. Effect of the size of the structure

As discussed above, there are many reasons to expect the quality of structures to deteriorate with increasing size. We set out to assess this by matching structures of different sizes (assessed by asymmetric unit volume) with a common subset, matching by resolution and deposition date but not by size. Again, the task is complicated by the non-uniformity of the size distribution. We followed a similar procedure as for resolution, choosing as the ‘cases’ a set of 355 structures from the peak of the distribution, with an asymmetric unit volume between 100 000 and 102 000 Å^3^, and choosing control sets each with 1/20 of the structures sorted by asymmetric unit volume.

**Figure 3:**
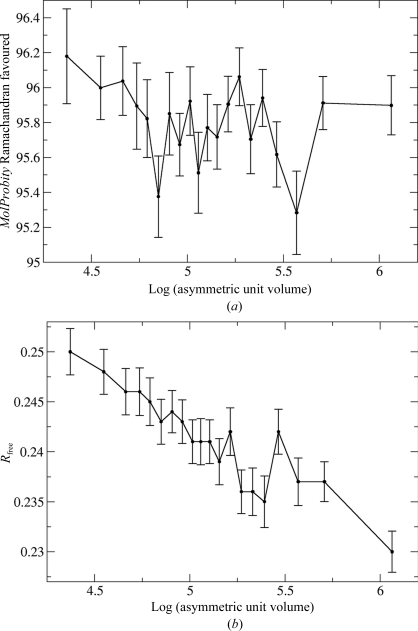
Variation in validation criteria with asymmetric unit volume, comparing structures with asymmetric unit volumes between 100 000 and 102 000 Å^3^ with matched structures in other volume ranges. (*a*) Percentage of residues in favoured regions of the Ramachandran plot, as assessed by *MolProbity* (Davis *et al.*, 2007 ▶). (*b*) *R*_free_.

The results, shown in Fig. 3 ▶, confounded our expectations. Once structures have been matched for other factors (such as resolution and date of deposition), there is no outstanding trend in most validation criteria. For example, Fig. 3 ▶(*a*) shows that there is no clear trend for the percentage of residues in the favoured regions of the Ramachandran plot as assessed by *MolProbity* (Davis *et al.*, 2007 ▶). One exception is the *R*_free_ value which, as shown in Fig. 3 ▶(*b*), decreases slightly but systematically with asymmetric unit volume. However, it should be noted that the range of variation is very small compared with that seen with resolution in Fig. 2 ▶(*b*). We suspect that this small systematic trend could arise from the increased frequency of noncrystallographic symmetry in structures with larger unit cells, a factor for which we have not yet accounted.

## 4. Case-control comparisons

### 4.1. Correlations with journal impact factor

Initially, we chose to study the effect of journal impact factor as a positive control, because there is a general perception in the crystallographic community that structures published in the highest impact journals are of lower average quality than those published in reputable but lower impact journals. There are reasons one might expect this to be true: publications in high-impact journals are usually in highly competitive fields where there is great pressure to be first, so that there might be a temptation to be satisfied with the refinement at an incomplete stage. Further, the editors and referees of such journals possibly focus more on the biological impact than the technical quality of submitted manuscripts. Finally, in the past three decades a disproportionally large number of (high-profile) structures that were subsequently shown to be seriously flawed have been published in high-impact journals (Brändén & Jones, 1990 ▶; Kleywegt, 2000 ▶; Davis *et al.*, 2008 ▶). Recent results by Brown & Ramaswamy (2007 ▶) appear to confirm that quality is negatively correlated with journal impact factor.

**Figure 4:**
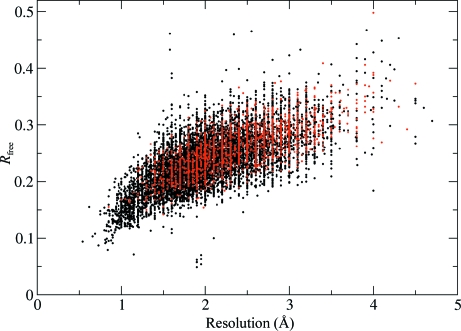
Scatter plot showing *R*_free_ as a function of resolution. Red points represent structures published in ‘high-impact’ journals, while black points represent structures published elsewhere.

Table 2 ▶ shows that structures published in high-impact journals indeed have poorer validation statistics than structures published elsewhere. However, a fair comparison has to account for the fact that structures published in high-impact journals are on average larger than those published elsewhere; as one might expect, they also diffract to lower resolution (Table 2 ▶). In fact, when ‘high-impact’ structures are compared with controls matched for resolution, asymmetric unit volume and date of deposition, the difference in average validation scores is greatly reduced. The scatter plot in Fig. 4 ▶ shows how the apparent average differences in *R*_free_ arise from a systematic difference in the distribution of resolution for structures published in high-impact and lower impact journals.

There are still small differences between the two sets of structures, but we have not accounted yet for other factors that may influence quality, such as the method of structure determination; for instance, a greater proportion of high-impact structures may be determined by experimental phasing rather than molecular replacement. Given the small size of the remaining differences, there is little evidence supporting the general perception that quality is systematically lower in the higher impact journals.

As noted above, we attribute our failure to reproduce the conclusions by Brown & Ramaswamy (2007 ▶) to the artefacts of the linear model they used in their statistical analysis. Looking at the data in Fig. 2 ▶(*c*), it is clear that a linear model would predict that lower resolution structures can be determined with better validation scores than we see with the case-control analysis of the data.

### 4.2. Structural genomics *versus* conventional structural biology

We carried out a preliminary survey, similar to this study, for presentation at the ICSG meeting in 2004. At that time, there was real concern that the pressure for productivity in structural genomics initiatives would result in lower standards and it had even been proposed that structure deposition in PSI-funded projects could be automatically triggered when the validation criteria passed some threshold.

The comparison is complicated by systematic differences in the goals of structural genomics and conventional structural biology. Indeed, because the goals differ between most of the structural genomics projects tabulated in TargetDB (Chen *et al.*, 2004 ▶) and the Structural Genomics Consortium (SGC; http://www.thesgc.com), these two structural genomics groups have been analyzed separately.

The results in Table 3 ▶ show that the average structural genomics structure is smaller, as one might expect, and diffracts to higher resolution. Without matching for resolution, asymmetric unit volume and date of deposition, structural genomics structures score slightly better on a number of validation criteria, such as *R*_free_, data completeness and percentage of residues in the favoured regions of the Ramachandran plot as assessed by *MolProbity* (Davis *et al.*, 2007 ▶). However, the case-matched control comparison shows that when comparing like with like the quality of structures determined in the different settings is much more similar.

Even with the case-matching, the structural genomics structures have slightly better Ramachandran scores, but this is likely to be influenced by another systematic difference. Particularly in the case of the SGC structures, the completeness of the model (assessed by comparing the residues contained within the PDB entry with the sequences given in the SEQRES records) is systematically lower. Many of the most poorly ordered residues would most likely be Ramachandran outliers if they were included in the model, so this confounds attempts to compare these sets of structures. (Note that an apparent variation in completeness could arise if groups differed in whether or not they included disordered residues arising from His tags or cloning artefacts, but the number of extra residues in tags is not sufficient to explain the differences found.)

It is important to emphasize that there is no community consensus on whether or not to include coordinates for poorly ordered parts of the structure. Within the Oxford site of the SGC at least, it has been decided that it is best to omit parts of the structure in which confidence is low (Frank von Delft, personal communication), but many other structural biologists prefer to include coordinates when there is even a weak indication of the polypeptide trace in the density.

## 5. Conclusions

Many factors are expected to influence the quality of crystal structures. Assessing which factors are important is complicated greatly by the fact that many of these factors are highly correlated (*e.g.* the size of the structure with the resolution of the data). By applying a case-control approach to comparing sets of structures, we have been able to establish that of the factors we evaluated, only the reported resolution and the date of deposition have a strong independent influence on the quality of structures. Surprisingly, once other factors have been controlled, there is very little dependence of most validation criteria on the size of asymmetric unit, whether the structure was determined as part of a structural genomics initiative or where the structure was published. The fact that some of these conclusions fly in the face of conventional wisdom demonstrates that the case-control approach is a valuable one.

The case-control approach could be generalized to apply to the assessment of individual structures simply by finding a reasonable number (say 100–200) of comparable controls in the PDB instead of one per structure. Applied to new depositions at the PDB, routine comparison of each entry at deposition time to a sample of 100–200 recent structures (up to 3 y old, perhaps) of similar resolution could be used for validation purposes. Thus, any models with unusual properties could be detected before they enter the structural archive.

Of course, a great deal of work remains to be done. We have not yet been able to evaluate the impact of other factors that are expected to influence quality. Most notably, these include the presence of noncrystallographic symmetry, the effects of twinning and anisotropy and the existence at the time of deposition of structures of close homologues at higher resolution.

However, it is worth emphasizing again that the diligent crystallographer should not be satisfied with doing as well as the average comparable structure, as it is probably possible to do better. We hope that the gradual improvement of structure quality over time will continue, so that the bar continues to be raised.

## Figures and Tables

**Table 1:** Space-group dependence of validation statistics Results are shown for the five space groups encountered most frequently in the data set. Values in parentheses are the estimated standard uncertainties of the means, computed as the sample standard deviation divided by the square root of the number of observations. Typical standard uncertainties for the *R* factors (not shown) are of the order of 0.05%. Mean *R* factors were computed for the subset of PDB entries for which they were reported. Note that the matched structures (‘mates’) are comparable to the cases, but crystallize in a different space group.

				Mean *R*_work_ (%)		Mean *R*_free_ (%)	
Set	No. in set	Mean resolution (Å)	Mean ASU volume (Å^3^)	Case	Mate	Case	Mate
All	38860	2.144 (0.003)	3.1 × 10^5^ (0.1 × 10^5^)	19.9	—	24.2	—
*P*2_1_2_1_2_1_	8918	2.062 (0.005)	3.0 × 10^5^ (0.2 × 10^5^)	19.6	19.6	23.9	23.8
*P*2_1_	5691	2.051 (0.006)	3.3 × 10^5^ (0.3 × 10^5^)	19.5	19.6	23.9	23.6
*C*2	3629	2.103 (0.008)	2.8 × 10^5^ (0.2 × 10^5^)	20.0	19.9	24.3	24.1
*P*2_1_2_1_2	2354	2.167 (0.010)	3.7 × 10^5^ (0.6 × 10^5^)	20.0	20.0	24.6	24.3
*C*222_1_	1981	2.197 (0.011)	3.2 × 10^5^ (0.2 × 10^5^)	20.2	20.2	24.4	24.5

**Table 2:** Dependence of validation statistics on journal impact factor Structures for which the primary literature reference is in *Nature*, *Science*, *Cell*, *Molecular Cell* or *EMBO Journal* (high impact) are compared with structures published in other journals (low impact) both for the entire set of low-impact structures and for structures selected as mates of the high-impact structures. Mean *R* factors are computed for the subset of PDB entries for which they were reported.

Criterion	Low impact	High impact	Low-impact mates
Resolution (Å)	2.12	2.36	2.36
ASU volume (Å^3^)	2.92 × 10^5^	4.32 × 10^5^	4.31 × 10^5^
*R*_work_	0.198	0.216	0.207
*R*_free_	0.240	0.259	0.253
Real-space *R* factor	0.138	0.169	0.151
*MolProbity* Ramachandran favoured (%)	95.5	93.1	93.8

**Table 3:** Dependence of validation statistics on setting for structure determination Structures determined as part of publicly funded structural genomics efforts (with entries in TargetDB; Chen *et al.*, 2004 ▶) or the Structural Genomics Consortium (SGC; http://www.thesgc.com) are compared with structures determined outside of these projects (non-SG). Mean *R* factors are computed for the subset of PDB entries for which they were reported.

	Non-SG	TargetDB		SGC	
Criterion	All	Case	Mate	Case	Mate
Resolution (Å)	2.15	2.06	2.06	2.04	2.04
ASU volume (Å^3^)	3.28 × 10^5^	1.80 × 10^5^	1.80 × 10^5^	1.74 × 10^5^	1.74 × 10^5^
*R*_work_	0.199	0.201	0.199	0.195	0.198
*R*_free_	0.243	0.239	0.239	0.237	0.239
Data completeness (%)	93.2	96.2	95.1	97.3	96.3
*MolProbity* Ramachandran favoured (%)	96.1	96.5	96.0	97.3	96.7
Model completeness (%)	96.1	94.1	95.3	90.4	95.0
